# Supplementary material for: Induced interpretation bias affects free recall and episodic memory bias in social anxiety
Source: PLoS One. 2023 Nov 16;18(11):e0289584. doi: 10.1371/journal.pone.0289584 (PMC10653471; doi:10.1371/journal.pone.0289584)
Supplement: Attachment — Submitted filename: response letter-22.12.11.docx [file pone.0289584.s002.docx]

Response to Peer Review Comments.

**Dear Professor Anindita Bhadra,**

**Thank you for your editorial letter regarding the manuscript entitled “Induced interpretation bias affects free recall and episodic memory bias in social anxiety”. We are very pleased with another opportunity for revising our manuscript. We have tried to do our best to address the concerns each reviewer raised. We have also revised our manuscript as per your advice below. Especially, we have updated statistical analyses using the generalized linear mixed models (GLMM) as you advised. I sincerely apologize for the delay in submitting the revised manuscript. I want to assure you that I took the necessary time to learn the statistical method you suggested, and to thoroughly re-analyze and derive the results to ensure their accuracy and validity. Thank you for your patience and understanding."**

Journal Requirements:

1. Please ensure that your manuscript meets PLOS ONE's style requirements, including those for file naming. The PLOS ONE style templates can be found at

https://journals.plos.org/plosone/s/file?id=wjVg/PLOSOne_formatting_sample_main_body.pdf and

<https://journals.plos.org/plosone/s/file?id=ba62/PLOSOne_formatting_sample_title_authors_affiliations.pdf>

**Response. we have revised the manuscript as you requested.**

2. Please provide additional details regarding participant consent. In the ethics statement in the Methods and online submission information, please ensure that you have specified what type you obtained (for instance, written or verbal, and if verbal, how it was documented and witnessed). If your study included minors, state whether you obtained consent from parents or guardians. If the need for consent was waived by the ethics committee, please include this information.

Once you have amended this/these statement(s) in the Methods section of the manuscript, please add the same text to the “Ethics Statement” field of the submission form (via “Edit Submission”).

For additional information about PLOS ONE ethical requirements for human subjects research, please refer to <http://journals.plos.org/plosone/s/submission-guidelines#loc-human-subjects-research>.

**Response. we have updated the method section to include detailed information about participant consent.**

On the day of the experiment, the participants completed the written consent form followed by the secondary screening survey.

3. Thank you for stating in your Funding Statement:

“This work was supported by the National Research Foundation of Korea (NRF) grant funded by the Korea government (MSIT) (No. 2018R1C1B5043272).”

Please provide an amended statement that declares *all* the funding or sources of support (whether external or internal to your organization) received during this study, as detailed online in our guide for authors at http://journals.plos.org/plosone/s/submit-now.  Please also include the statement “There was no additional external funding received for this study.” in your updated Funding Statement.

Please include your amended Funding Statement within your cover letter. We will change the online submission form on your behalf.

**Response. as per your suggestion, we have changes to the Funding statement, whihch is now updated as follows:**

“This work was supported by the National Research Foundation of Korea (NRF) grant funded by the Korea government (MSIT) (No. 2018R1C1B5043272). There was no additional external or internal funding received for this study”.

4. Thank you for stating the following in the Acknowledgments Section of your manuscript:

“This work was supported by the National Research Foundation of Korea (NRF) grant funded by the Korea government (MSIT) (No. 2018R1C1B5043272). “

We note that you have provided additional information within the Acknowledgements Section that is not currently declared in your Funding Statement. Please note that funding information should not appear in the Acknowledgments section or other areas of your manuscript. We will only publish funding information present in the Funding Statement section of the online submission form.

Please remove any funding-related text from the manuscript and let us know how you would like to update your Funding Statement. Currently, your Funding Statement reads as follows:

“This work was supported by the National Research Foundation of Korea (NRF) grant funded by the Korea government (MSIT) (No. 2018R1C1B5043272).”

Please include your amended statements within your cover letter; we will change the online submission form on your behalf.

**Response. we have removed the Funding statement, including any acknowledgements of funding sources, from the manuscript. Please update the Funding statements below accordingly.**

**“This work was supported by the National Research Foundation of Korea (NRF) grant funded by the Korea government (MSIT) (No. 2018R1C1B5043272). There was no additional external or internal funding received for this study”.**

5. In your Data Availability statement, you have not specified where the minimal data set underlying the results described in your manuscript can be found. PLOS defines a study's minimal data set as the underlying data used to reach the conclusions drawn in the manuscript and any additional data required to replicate the reported study findings in their entirety. All PLOS journals require that the minimal data set be made fully available. For more information about our data policy, please see http://journals.plos.org/plosone/s/data-availability.

"Upon re-submitting your revised manuscript, please upload your study’s minimal underlying data set as either Supporting Information files or to a stable, public repository and include the relevant URLs, DOIs, or accession numbers within your revised cover letter. For a list of acceptable repositories, please see http://journals.plos.org/plosone/s/data-availability#loc-recommended-repositories. Any potentially identifying patient information must be fully anonymized.

Important: If there are ethical or legal restrictions to sharing your data publicly, please explain these restrictions in detail. Please see our guidelines for more information on what we consider unacceptable restrictions to publicly sharing data: http://journals.plos.org/plosone/s/data-availability#loc-unacceptable-data-access-restrictions. Note that it is not acceptable for the authors to be the sole named individuals responsible for ensuring data access.

We will update your Data Availability statement to reflect the information you provide in your cover letter.

**Response. we have uploaded the dataset as Supporting information.**

6. Please ensure that you include a title page within your main document. You should list all authors and all affiliations as per our author instructions and clearly indicate the corresponding author.

**Response. we have completed it as per your instructions**

7. Please include a separate caption for each figure in your manuscript.

**Response. we have included separate captions for each of the figures, namely, figure 1 and figure 2.**

8. We note you have included a table to which you do not refer in the text of your manuscript. Please ensure that you refer to Table 1 in your text; if accepted, production will need this reference to link the reader to the Table.

**Response. we have included descriptions of Table 1, Table 2, and Table 3 in the manuscript.**

Additional Editor Comments (if provided):

This is an interesting piece of work, and I would like to see this published. As you would see, the two expert reviewers have very opposing views on the manuscript. I personally feel that the manuscript needs editing. For example, there are abbreviations being used, without explaining what they are. Some of the references are not in order, as Reviewer 1 has already pointed out. I would also suggest that a flowchart is added to explain the steps of the study. This would provide a good overview to the readers. Further, I would recommend using a generalized linear model, instead of the mixed-model ANOVA for the analysis.

**Response: "In response to your helpful comments, we have made two significant changes to the manuscript. Firstly, we have included a flowchart as Figure 1. Secondly, we have used a GLMM in place of a mixed model ANOVA to analyze the SRT and free recall and past episodic memory bias data. Thank you for your valuable feedback."**

**Comments to the Author**

5. Review Comments to the Author

Please use the space provided to explain your answers to the questions above. You may also include additional comments for the author, including concerns about dual publication, research ethics, or publication ethics. (Please upload your review as an attachment if it exceeds 20,000 characters)

Reviewer #1: Overall, the manuscript needs significant improvement in terms of clarity and writing coherence. Also, there are some grammatical and writing errors that getting help from a native English speaker who is familiar with psychological research to proofread the work would help with that.

Abstract

1. Please mention the number of participants in the abstract.

Response. As per your comments, we have updated the number of participants in the abstract. Thank you for your valuable feedback.

The combined effect of each cognitive bias is known to play a causal role in the etiology and maintenance of social anxiety. However, little is known about how each type of bias acts during social anxiety. The present study aimed to investigate whether experimentally induced interpretation bias using the cognitive bias modification (CBM) paradigm would influence free recall and episodic memory biases in a Korean sample. A total of 61 participants were randomly assigned to either a positive (*n* = 30) or negative (*n* = 31) CBM group.

Intro
1. I suggest that authors summarize/cut some of the social anxiety disorder prevalence-related information in the first paragraph of the introduction to make this part more to the point.

Response. Thank you for your helpful feedback. We have summarized the first paragraph related to social anxiety disorder.

2. Please check the in-text citations as currently some of them seems incorrect, for example, Amir, Foa (7).

Response. We have revised the in-text citations throughout the manuscript in consultation with the editorial office.

3. In line 30, in describing the Amir et al (7) study, the authors didn’t clarify whether the social anxiety groups in this study were clinical participants or subclinical individuals with elevated social anxiety. Please specify for this study and throughout the manuscript what is meant by “group with social anxiety”.

Response. Thank you for your comments. We have clarified the characteristics of the participants as follows:

For example, Amir, Foa and Coles [6] presented ambiguous social or non-social situations to three groups; clinical groups of people with generalized social phobia and obsessive-compulsive disorder, as well as a control group without social anxiety.

4. The definition of the CBM-I in lines 41-43 is not clear, please provide a more informative definition.

Response. Thank you for your feedback. We have provided a clearer description of CBM-I.

Cognitive Bias Modification of interpretation (CBM-I) aims to positively correct negative biases in individuals with anxiety or depression, by providing repetitive computer-based tasks for interpreting ambiguous social situations in a more flexible and positive way [9]. Participants can complete the training task at home or wherever they prefer [10].

5. It is unclear what is meant by “distortion of memory” in line 76. Please specify.

Response. We added detailed information that reflects the result of the experiment.

On recalling the scenarios, the social anxiety groups tended to report a higher percentage of false errors with emotionally negative content compared with the control group.

6. In line 79, it is unclear what the pronoun “them” is referring to.

Response. We revised ‘them’ into ‘ambiguous social scenarios.

One group of participants was trained to interpret ambiguous social scenarios positively, and the other, negatively.

7. It is better to avoid using the word “prove” for psychological hypotheses.

Response. We have updated the word in our manuscript from “prove” to “supported” for all hypotheses throughout the manuscript. For instance,

These results partially supported Hirsch and Clark’s [1] combined cognitive biases hypothesis that interpretation bias affects memory.

8. The paragraph that sums up the rationale for the study is not clear, and needs to be re-written. It seems somewhat disconnected from previous paragraphs, and some parts of it are difficult to follow and understand. For example, I suggest that authors describe the auditory method of reading the scenarios before this paragraph. Because this is an important point in the design of the current study and the first time it appears about the end of the introduction without it being sufficiently described before.

Response. Thank you for your feedback. We have added a more detailed explanation of CBM-I, including the use of auditory imagery in the intervention, to the manuscript.

Cognitive Bias Modification of interpretation (CBM-I) aims to positively correct negative biases individuals with anxiety or depression, by providing repetitive computer-based tasks for interpreting ambiguous social situations in a more flexible and positive way [9]. Participants can complete the training task at home or wherever they prefer. Mathews and Mackintosh [10]found that participants in the positive CBM-I group showed significantly reduced levels of anxiety as well as increased positive interpretation bias compared with participants in the negative CBM-I group, confirming the causal relationships between cognitive bias and mood. Mathews and Mackintosh [10] argued that CBM-I, which induces positive interpretations in social anxiety, may have therapeutic implications that reduce anxiety caused by negative interpretation bias. Indeed, many prior studies with the subclinical population with social anxiety symptoms have since demonstrated iterative CBM-I training can modify interpretation bias for social situations and then affect the change of symptoms [9, 11, 12]. Previous studies have explored the optimal mode of delivering CBM-I intervention that involve short stories based on daily life experiences. It has been hypothesized that imagining scenarios mentally is more effective in influencing mood compared to verbal reasoning. Indeed, several studies have demonstrated that auditory CBM-I that utilizes imagery has a greater impact on mood and interpretation biases compared to visually presented CBM-I intervention [13, 14] [15].

Method

1. The first time you use an abbreviation, spell out the full term and put the abbreviation in parentheses.

Response. We have thoroughly checked and spelt out the full name.

A total of 114 undergraduate and graduate students completed an online screening survey using the Center for Epidemiological Studies-Depression (CES-D), Social Avoidance Distress Scale (SADS), and Brief version of Fear of Negative Evaluation Scale (B-FNE).

2. Please provide more information for each questionnaire, what the questionnaire was designed to measure, if it has been reported as a valid and reliable measure by previous researchers, and if the psychometric properties of the Korean version of the questionnaire have been examined.

Response. In response to your comments, we have included additional information about he the purpose of each questionnaire, its previous use as valid and reliable measure, and whether its psychometric characteristics were evaluated in the Korean version of the questionnaire.

**Questionnaires**

***Center for Epidemiological Studies-Depression (CES-D)****.*

The CES-D [23, 24] was utilized during the screening phase to measure the degree of depression experienced in the previous week, using a four-point Likert scale (0 = extremely rare to 3 = most likely). It has demonstrated strong psychometric properties, including a high internal consistency coefficient (Radloff [23] Cronbach’s α = .85; Cho and Kim [24] Cronbach’s α = .91) and satisfactory test-retest reliability (Radloff [23] r = .57; Cho and Kim [24] r = .68). Our study yielded a Cronbach's α of .70.

***Social Avoidance and Distress Scale (SADS)***.

The SADS [25, 26] was used in the screening phase to evaluate the anxiety and inclination of participants to avoid social situations on a five-point Likert scale (ranging from 1 = do not agree at all to 5 = strongly agree). It has good psychometric properties, including a high internal consistency coefficient (Lee and Choi [26] Cronbach’s α = .92) and satisfactory test-retest reliability (Watson and Friend [25] r = .68; Lee and Choi [26] r = .88). Our Cronbach's α was .88.

***Brief Version of Fear of Negative Evaluation Scale (B-FNE)*.**

The B-FNE [25-27] was used during the screening phase to assess the fear of being negatively evaluated by others, which is a key characteristic of social anxiety. Response were given on a five-point Likert scale ranging from (not at all) to 5 (very much). The questionnaire has been reported to have good psychometric properties, including high internal consistency coefficient (Lee and Choi [26]; Leary Lee and Choi (26), [27] Cronbach’s α = .90) and good test-retest reliability (Watson and Friend [25] r = .78; Lee and Choi [26] r = .80; Leary [27] r = .75). Our study obtained a Cronbach’s α of .89.

***Visual Analogue Scale (VAS)***.

To assess participants’ state mood, they were asked to rate their mood before and after the training and the first filler task. The VAS was used as a self-report measure of current emotions and pain. The VAS is a simple tool that allows individuals to rate their subjective state [28] by marking an 'X' on a horizontal bar ranging from 0 (not at all) to 10 (extremely) for negative (sadness) and positive (happiness) emotions. This scale is ideal for self-reporting as the response burden is low [29].

***Six-item short version of the state-trait anxiety inventory (STAI-6)***.

STAI-6 [30] is the shorter version of the State-Trait Anxiety Inventory-State (STAI-S) [31]. It assesses the current level of anxiety using a four-point Likert scale (1 = not at all to 4 = strongly agree). Compared to STAI-S, it is a concise, reliable, and valid measure that is sensitive to changes in anxiety [30, 32]. Previous studies have reported high internal consistency coefficient (Tluczek, Henriques [32] Cronbach’s α ranging from .74 to .82). In our study, the Cronbach’s α was .74 before training, .80 after training, and .77 after the filler tasks.

3. The similarity rating task description is not clear enough. It is not clear what the authors meant by the "title of scenarios". Moreover, the order of describing different parts of the task is not optimal, for example, the authors first wrote that the participants were asked to complete the incomplete word in the last phrase of the scenario, and then the scenarios. Generally, this section needs to be re-written more clearly with providing an example trial/scenario to help the readers to follow and understand the task.

Response. We have revised and rewritten the description of the SRT with an example.

4. What was the neutral interpretation question in the SRT task? Please provide more information on that.

Response. We have provided the rewritten description of the SRT with an example.

5. In the recognition phase of the SRT how many trials/scenarios were presented for recognition? Was it the case that each scenario's title was presented with all four types of sentences? Please clarify.

Response. We have provided a more detailed description of the SRT. This includes an example to help illustrate how the task is performed by participants.

6. Please explain the indices that were computed from the SRT task in the same section you described the task.

Response. The indices used in the final analysis have been included at the end of the SRT description.

***Similarity Rating Task (SRT).***

The SRT was used in this study to assess the participants’ interpretation bias after CBM training. The procedure and format of the SRT were based on the previous studies [35, 36]. The SRT comprises two phases: encoding and recognition. In the encoding phase, below the title of each story, participants were presented with 10 scenarios, each with an ambiguous and neutral descriptions ending in a word fragment completion, followed by a neutral comprehension question (‘yes or no’ answer) regarding the content of each description. An example is as follows.

<Presentation of liberal arts class>

You give a presentation in a liberal arts class.

After the presentation, some people ask you questions.

As you answer the last question, the classroom goes sil__nt.

Did you answer the question?

[yes or no]

In the following recognition phase, each scenario’s title was presented along with four types of sentences in random order: (1) positive sentence related to the scenario (target positive; i.e., *people are happy with my answer*), (2) negative sentence related to the scenario (target negative; i.e., *people are so frustrated with my answer*), (3) positive sentence unrelated to the scenario (foil positive; i.e., *you finish your presentation on time*.), and (4) negative sentence unrelated to the scenario (foil negative; i.e., *you don't finish your presentation on time*). Participants were asked to rate how similar each sentence was to the scenario they had read in the encoding phase on a four-point Likert scale (1 = very different; 2 = fairly different; 3 = fairly similar; 4 = very similar). The target sentences were either positive or negative interpretation related to the social situation scenario, the foil sentences were generally positive or negative [37]. The four types of sentences were fixed for each scenario and were presented in a random order. This study included 40 trials, with each scenario having four different trials consisting of a target positive, a target negative, a foil positive, and a foil negative interpretation.

The SRT was conducted using the E-prime 2.0 software and displayed on a 16-inch laptop screen with a white background and navy-colored Arial font. Before actual test, participants completed five practice trials. The mean score for each type of the sentence (target positive, target negative, foil positive, and foil negative) was calculated and used in the final analysis.

7. In the free recall task description, the authors named "the new content that did not apply to the scenarios or recognition task sentences" as intrusions! I don't think we can call these incorrect memories as intrusions. Intrusions are involuntarily thoughts and images that are irrelevant and interrupting to the ongoing activity, while in the recall task participants themselves were actively trying to remember content related to the scenarios, therefore even if they remembered something incorrect these could not be named intrusions.

Response. We appreciate your feedback. The scoring procedure for intrusions was informed by previous research conducted by Salemink, Hertel, and Makintosh (2010), as well as Joormann, Waugh, and Gotlib (2015). Specifically, Salemink, Hertel and Mackintosh (2010) defined intrusions as “terms that had not been presented originally”, and we adopted this definition after consulting with Joormann prior to the study. During the SRT recall task (i.e., both encoding and recognition phases), participants occasionally reported irrelevant and distracting information. Whether these intrusions were intentional or unintentional remains unclear. However, we scored intrusions according to the definition established in previous research, namely as irrelevant content that was not related to the SRT descriptions.

8. The difference between what is defined as “intrusions” and "new ideas" is not clear, and it seems these two categories overlap largely, which is a methodological issue.

Response. Thank you for the clarification. Based on the scoring system used by Salemink, Hertel, and Mackintosh (2010) and Joormann, Waugh, and Gotlib (2015), intrusions were defined as any new terms or ideas that were not originally presented in the SRT task. On the other hand, new autobiographical memory ideas were categorized separately from intrusions, namely new ideas.

Results

1. The authors only analyzed the second part (recognition phase) of the SRT task, while the first part that asked participants to complete the last word of the ambiguous neutral sentences has not been analyzed. It is possible that the CBM-I groups significantly differed in interpreting the sentences of the first phase of the SRT task.

Response. Thank you for the suggestion. In the first part of the SRT, participants were presented with a script that included an empty word, which they were required to fill in (e.g., "As you answer the last question, the classroom goes sil__nt"), followed by a question to confirm their understanding of the script (e.g., "Did you answer the question? Yes or No?"). We analyzed the responses of the first part of the SRT and found no significant differences between the two groups. The correct response rate was almost identical for both groups.

2. Please specify the dependent variable for each ANOVA analysis.

Response. Thank you for the feedback. We have revised the manuscript and added a specification of the dependent variable for each ANOVA analysis.

**The effects of CBM-I on state mood**

To examine the effects of CBM-I on participants’ mood states, we conducted a 2 (group: positive CBM-I, negative CBM-I) × 2 (time: Pre, Post) × 2 (valence: happiness, sadness) repeated ANOVA (dependent variables: pre- and post-levels of sadness and happiness).

**The effects of CBM-I on interpretation bias**

To investigate the effects of CBM-I on interpretation bias, a 2 (group: positive CBM-I, negative CBM-I) × 2 (sentence type: target, foil) × 2 (valence: positive, negative) mixed-model ANOVA was conducted on SRT (dependent variables: target positive, target negative, foil positive, foil negative).

**Free recall task**

The aim of the present study was to investigate whether CBM-I training affected the participants’ memory bias. We conducted a 2 (group: positive CBM-I, negative CBM-I) × 3 (valence: positive, negative, neutral) mixed model ANOVA each for the total number of intrusions and content (dependent variables related to intrusion included each positive, negative and neutral intrusion, while the dependent variables related to content included each positive, negative and neutral content).

**Past thinking task**

In addition to the memory bias for SRT scenarios, we investigated whether memory bias could be observed in past memories. Specifically, a 2 (group: positive CBM-I, negative CBM-I) × 3 (valence: positive, negative, neutral) mixed-model ANOVA was performed for the total number of positive, negative, and neutral memories [41] (dependent variables: the total number of each positive. negative memories, neutral episodic memories).

3. For the free recall task, it is unclear why the authors based their main analyses on intrusion that they themselves defined as "reporting of new content that did not apply to the scenarios or recognition task sentences". What were the reasons that the authors did not use the degree to which participants of each group remembered accurate negative or positive material?

Response. Thank you for your comments. The scoring procedure for intrusions was informed by previous research conducted by Salemink, Hertel, and Makintosh (2010), as well as Joormann, Waugh, and Gotlib (2015). Specifically, Salemink, Hertel and Mackintosh (2010) defined intrusions as “terms that had not been presented originally”, and we adopted this definition after consulting with Joormann prior to the study. During the SRT recall task (i.e., both encoding and recognition phases), participants occasionally reported irrelevant and distracting information. Whether these intrusions were intentional or unintentional remains unclear. However, we scored intrusions according to the definition established in previous research, namely as irrelevant content that was not related to the SRT descriptions.

We did not come across any explicit criteria defining the accuracy of positive or negative material recalled by the participants. Rather, as we addressed in our previous responses, we classified the emotional valence of all the recalled content as positive, negative, or neutral. As an illustration, a participant's statement such as "I was nervous and did my presentation awkwardly, but I managed to complete it well and received good feedback" was regarded as positive, despite containing a negative sentence. However, we did not evaluate the accuracy of the participants' responses.

4. For the second ANOVA in the Free recall task analysis, it is unclear what exactly meant by "content". Was it everything that participants remembered or a specific part of what they remembered?

Response. Thank you for your comments. In the free recall task, as mentioned in the manuscript, we coded the valence of all the content recalled by participants as positive, negative, or neutral. For example, a statement such as “I was nervous and did my presentation awkwardly, but I managed to completed it well and received good feedback” which included a negative sentence but had overall positive content, was coded as positive.

5. Why the authors controlled for the role of gender in the ANOVA analysis for past thinking task, while the same had not been done for previous analyses?

Response. Thank you for your feedback. As there was a significant difference in gender for past thinking task, but not for other tasks, we conducted a follow-up analysis by controlling for gender. However, the results of the follow-up analysis remained unchanged.

Discussion

1. The authors wrote that “a part of the combined cognitive biases hypothesis” was confirmed. What part of this hypothesis they exactly referring to? I think they meant the combined cognitive biases hypothesis for interpenetration bias and memory bias, so if this is the case, specify that instead of saying "a part" that is vague and unclear!

Response. Thank you for feedback. We have now provided specific information on which component of the combined cognitive bias is supported by our findings.

The main purpose of the present study was to examine whether experimentally induced interpretation bias would affect biases in memory in Korean samples. In doing this, the present study used CBM scenarios that were auditory-specific and with a focus on social anxiety symptoms. The main results showed that interpretation biases can be induced, and induced interpretation biases resulted in training congruent state mood and memory biases on both free-recall memory and autobiographical memory. These results supported Hirsh and Clark’s [1] combined cognitive biases hypothesis that interpretation bias affects memory.

2. Line 401, control group are described as "healthy people without any social anxiety and depression". The sentence seems not true given the dimensional nature of social anxiety and depression.

Response. Thank you for the feedback. We have revised the original phrase ‘without any social anxiety and depression’ to ‘with lower levels of social anxiety and depression’.

The reason for this finding might be that participants in the present study, unlike in prior studies, are healthy people with lower levels of social anxiety and depression. In previous works (10, 18), the participants were likely to be recruited without a screening process and so included a mix of healthy people as well as those who tend to be depressed and socially anxious.

Reviewer #2: Well written and without error. I would ask the authors to make one more check that all word spacing is correct, all terms are properly expanded (particularly DSM-5 on page 1, sentence 1) and the use of references in the paper are correct as per the journal requirements. I use the ACS style guide more than any other reference and yours may be perfectly correct, but simply different than what I am familiar with.

Response. Thank you for your feedback. We have made the necessary changes to the reference citation to adhere to the Plos style guidelines.
