# Supplementary material for: Induced interpretation bias affects free recall and episodic memory bias in social anxiety
Source: PLoS One. 2023 Nov 16;18(11):e0289584. doi: 10.1371/journal.pone.0289584 (PMC10653471; doi:10.1371/journal.pone.0289584)
Supplement: Attachment — Submitted filename: response letter-23.5.23.docx [file pone.0289584.s003.docx]

Response to Peer Review Comments.

**Dear Professor Anindita Bhadra,**

**Thank you for your editorial letter regarding the manuscript entitled “Induced interpretation bias affects free recall and episodic memory bias in social anxiety”. We are very pleased with another opportunity for revising our manuscript. We have tried to do our best to address the concerns each reviewer raised. We have also revised our manuscript as per your advice below. Thank you for your patience and understanding."**

Reviewer #1: The authors have done an excellent job of addressing the comments and significantly improving the new version of the manuscript. Congratulations to the authors for conducting this interesting study. However, there is one major issue and two minor issues that I would like to raise regarding the new version of the work. I believe that after addressing the remaining issues, the manuscript will be ready for publication.

Major issue:

There is a misinterpretation of the results related to the CBM-I training effects. In the section “The effects of CBM-I on interpretation bias” the authors report: "Participants in the positive training group rated higher similarity for positive sentences than for negative sentences (mean estimate = 0.65, Confidence Interval = 0.4504~0.8663), whereas the negative group did not." The authors then summarize and conclude that “To summarize, participants in the positive training group showed positive interpretation bias regardless of sentence type (target and foil), agreeing with the trained valence, whereas participants in the negative training group did not show any effect of training”.
This is a problematic interpretation of the finding. There was no assessment of baseline interpretation biases before the CBM-I training in the current study, which makes it impossible to conclude that the negative CBM-I was not successful in influencing Interpretation Biases (IB) or that the positive CBM-I was successful in changing participants’ IB. This might be the case that all participants had a significantly more positive than negative IB at the baseline, especially that the researchers recruited participants with low levels of social anxiety for their study. If that is true (which we don’t know due to a lack of baseline assessment of IB), the negative CBM-I was indeed effective in reducing participants’ positive interpretation biases to a level that no significant difference was found between positive and negative interpretation biases in the negative group. Also, it would be the case that because participants already had more positive than negative IB, the positive CBM-I either did not impact their IBs or slightly increased them. Therefore, this cannot be concluded that the positive or negative CBM-I trainings were successful or not in changing participants’ interpretation biases as we don’t know participants’ initial baseline IB patterns. Although given that participants were randomly assigned to the CBM-I groups, it is very likely that their baseline IB were similar, and it can be concluded that their significant difference after the CBM-I training is due to the training, but still we don’t know if both groups had more positive than negative IB before the CBM-I training (in that case negative CBM-I was effective), or more negative than positive IB (in that case positive CBM-I was effective), or equal positive and negative IB.
Likewise, if authors hypothesize and expect that participants in the negative CBM-I show more negative than positive IB and those in the positive CBM-I show more positive than negative IB, they can say that these findings did not fully supported their hypothesis, but it is still incorrect to conclude that positive CBM-I was successful in changing IB and negative CBM-I failed to change participants IB for the same reason that I explained earlier.
The same problem can be seen in the discussion section too where the authors wrote:
“There was no effect of CBM-I training on interpretation bias in the negative group….”
They also write: “Second, the positive group revealed a positive interpretation bias in both the target and foil sentences, whereas the negative group did not show any difference before and after CBM training”. It is important to note that in this sentence, they mention “before and after CBM training,” but the interpretation bias assessment occurred only after the CBM-I training in the study.

I think this problem in the interpretation of the effects of the CBM-I training is very important and needs to be addressed throughout the manuscript.

Response.
Thank you for your insightful comments and for bringing attention to the issue regarding the interpretation of the effects of the CBM-I training in our study. We appreciate your valuable feedback and have taken your points into careful consideration.

We acknowledge the limitation of our study in not conducting a baseline assessment of interpretation biases (IB) prior to the CBM-I training. As you rightly pointed out, the absence of baseline data makes it challenging to draw definitive conclusions about the effectiveness of the positive and negative CBM-I interventions. We have incorporated this limitation into our discussion, emphasizing the need for future research to include baseline assessments to provide a more comprehensive understanding of the impact of CBM-I interventions on IB.

On the other hand, it is important to note that participants in our study were randomly assigned to the positive and negative training groups, which helps minimize initial differences between the groups. Random assignment increases the likelihood of similarity in baseline IB patterns. Thus, while we acknowledge the limitations, the observed differences in IBs between the groups can be attributed to the training itself. Furthermore, it is crucial to clarify that our study focused on the effects of the CBM-I training on participants' interpretation biases within the specific context of the study session. We did not explicitly aim to assess long-term changes in interpretation biases. The reported results reflect the immediate impact of the CBM-I training, indicating that the positive training group showed a positive interpretation bias consistent with the trained valence, while the negative training group did not exhibit a significant effect of training on their interpretation biases. While we understand that this conclusion does not capture the entire complexity of participants' IB patterns, it provides valuable insights into the immediate impact of the CBM-I training within the study session.

Regarding the specific goals and hypotheses of our study, we agree that it is important to clarify that our study did not explicitly aim to demonstrate the success or failure of the interventions in changing IB. Our primary focus was on exploring the interaction between interpretation biases and memory biases in a single session. Therefore, the conclusions we drew were within the context of our specific research goals and hypotheses. In response to your feedback, we have made revisions to the manuscript to address the issue you raised regarding the comparison between pre-training and post-training IB assessments. We have explicitly stated that our assessment of IB occurred only after the CBM-I training and have avoided making any statements that imply a comparison between pre-training and post-training IB. We have also incorporated the limitation you suggested regarding the lack of baseline assessment of interpretation bias in the final version of the manuscript.

Overall, we appreciate your valuable feedback and believe that your suggestions have significantly enhanced the accuracy and clarity of our interpretations. We have carefully addressed the issues you raised and made the necessary revisions throughout the manuscript.

Thank you for your time and guidance in helping us strengthen our study.

P 22

Finally, we acknowledge that the absence of a baseline assessment of interpretation bias is a limitation of our study. Therefore, we urge caution in interpreting the results regarding the effect of CBM-I training on interpretation bias. Despite the random assignment of participants to the positive and negative training groups, we cannot disregard the possibility that there might have been initial differences in positive and negative interpretation tendencies between the groups. To gain a more comprehensive understanding of the impact of CBM-I training on interpretation biases, future research should consider incorporating baseline measurements. This would enable a clearer assessment of the effectiveness of CBM-I interventions in shaping interpretation biases.

Minor issues
1- Please specify the name of the biases in the first two sentences of the abstract i.e. interpretation and memory. Currently, the way the abstract starts talks about "each bias" and "each type of bias" which seems not clear and can include any type of different bias.

Response. Thank you for your helpful comments. We have revised the first two sentences of the abstract to provide clarity regarding the specific biases under consideration. The revised sentences now specify interpretation, attention, and memory biases.

The combined effect of each cognitive bias, interpretation, attention, and memory bias, is known to play a causal role in the etiology and maintenance of social anxiety. However, little is known about how each type of bias (i.e., interpretation, memory bias) acts during social anxiety.

2- The order of two sentences in the introduction needs to be changed. Specifically, the sentence that starts with "Mathews and Mackintosh [10] found that participants in the positive CBM-I group...." seems to belong after its next sentence i.e. "Mathews and Mackintosh [10] argued that CBM-I, which induces positive interpretations in social anxiety, may have ..." not before it.

Response. Thank you for your valuable feedback. We have revised the order of the sentences in the introduction as per your suggestion.
